# A Translational Review of Mechanisms of Effectiveness of Photobiomodulation on Somatosensory Neurons and the Peripheral Nervous System—From Molecular Mechanisms to Clinical Applications in Medicine and Dentistry

**DOI:** 10.3390/cimb48070695

**Published:** 2026-07-09

**Authors:** Roberta Chow, Patricia Armati

**Affiliations:** Brain and Mind Centre, Faculty of Medicine and Health, University of Sydney, 94 Mallet Street, Camperdown, NSW 2050, Australia; patricia.armati@sydney.edu.au

**Keywords:** analgesia, dental anesthesia, dorsal root ganglion, photobiomodulation (PBM), fast axonal flow (FAF), pain, nociceptor, somatosensory neuron, mitochondrial membrane potential

## Abstract

This review aims to provide a translational link between clinical evidence in the application of photobiomodulation for treatment of painful conditions in medicine and dentistry and neurophysiological effects of photobiomodulation therapy (PBMt). PBMt is gaining increasing acceptance as a therapeutic modality for pain management, particularly within dental practice. However, a clearer understanding of its mechanisms of action remains essential for broader clinical adoption and integration into mainstream healthcare. Central to the therapeutic effects of PBMt for pain is its interaction with neurons, which possess unique structural and functional characteristics. Advances in our understanding of the cellular architecture of dorsal root and trigeminal ganglion neurons highlight the importance of the stem axon and axon initial segment (AIS), a specialized region adjacent to the axon hillock that is now recognized as the principal site of action potential initiation. These developments have important implications for understanding the biological effects of PBMt and its clinical application. This review synthesizes evidence demonstrating that PBM influences cytoskeletal organization, mitochondrial structure and function, and intracellular signaling pathways, with downstream effects on neuronal excitability and nerve conduction. By integrating findings from cellular, neurophysiological, and clinical studies, the review examines how these mechanistic effects may contribute to pain modulation and analgesia.

## 1. Introduction

The aim of this review is to synthesize data from PBM studies of molecular responses in single cultured neurons; in vivo and in vitro nerve studies and present a neurally based hypothesis for the modulation of pain in medicine and in dentistry. Pain remains a problematic and universally important clinical challenge with treatments for acute and chronic pain varied as to their effectiveness. Among treatments, light as photobiomodulation therapy (PBMt) is increasingly used. Although it is accepted that the retina responds to light at varying wavelengths and that Vitamin D is best obtained from sunlight, the idea of using light for pain remains outside mainstream medicine’s accepted practice though is becoming more widely used in standard dental therapy. While there are well-designed clinical trials showing PBMt’s effectiveness for painful musculoskeletal conditions [1,2,3] and, in dentistry, for pre-emptive anesthesia [4,5], the mechanisms of its effectiveness are still not well understood.

Clinically, PBMt is delivered to sites of pathology or over nerves at specific parameters including wavelength, dose, time protocol, area of delivery and more. As pain is mediated through the somatosensory nervous system it is logical to focus on the response of this system to laser irradiation (LI) to develop an understanding of how pain can be modulated. Reference to the unique and complex anatomy of peripheral nervous system (PNS) first-order neurons is central to understanding how PBM affects neurons which transduce noxious stimuli from the periphery into pain sensation in the brain. Recent research demonstrating the unique anatomy of these neurons highlights the important function of the axon initial segment (AIS) and the complexity of the T junction, the bifurcation forming the distal and proximal axons [6,7].

Our objective is to identify cellular and molecular mechanisms through which PBM affects neuronal function, to evaluate evidence for PBM-induced modulation of nociceptive signaling, to assess the translational relevance of experimental findings for clinical pain management and dental anesthesia and to identify gaps in current knowledge and priorities for future research.

## 2. Materials and Methods

The studies cited in this review were selected because they contribute mechanistic, neurophysiological, or clinical evidence relevant to understanding the effects of PBM on somatosensory neurons and pain processing. They provide a logical progression of studies from molecular to cellular and to whole nerve function in animal models and clinical studies across multiple levels of biological organization. Individual studies were selected for their biological relevance and to provide a coherent framework for a neurophysiological hypothesis in contrast to a systematic review of the literature. The studies represent well-established experimental paradigms, direct neurophysiological observations, or controlled clinical investigations that provide substantial evidence.

Such an approach is aligned with contemporary systems biology in which complex biological responses emerge from interactions across multiple scales rather than from isolated observations within a single experimental domain [8]. The present review therefore seeks to evaluate the coherence of these interconnected observations rather than to quantify effect size or provide an exhaustive systematic assessment of all published studies.

## 3. The Importance of Peripheral Nervous System Organization

**Figure 1:**
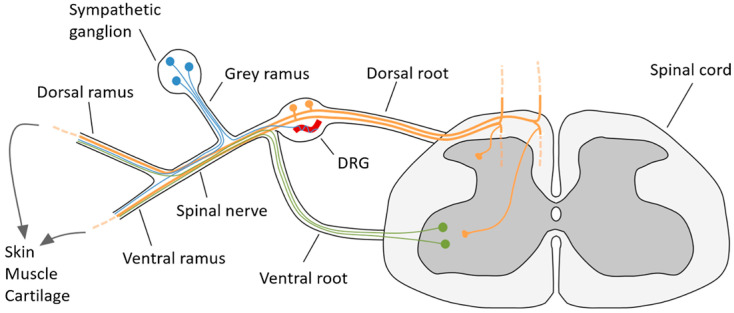
Diagram indicating sympathetic and central nervous system involvement in peripheral nerve organization. Orange—DRG neurons with cell bodies, proximal and distal axons. Green—Motor neurons. Blue—sympathetic neurons. Adapted with permission from Ref. [6], Progress in Neurobiology. P 87. Elsevier.

The Peripheral Nervous System (PNS) is made up of somatosensory first-order neurons however peripheral nerves include not only afferent axons transmitting sensory information to the Central Nervous System (CNS) but also motor axons arising from ventral horn cells of the spinal cord and sympathetic axons. The sensory nerve cell bodies lie within the DRG- and PNS-related ganglia such as the trigeminal nerve ganglia in humans and are of particular interest in understanding how noxious and other PNS stimuli are transmitted and perceived as pain (Figure 1).

### 3.1. Dorsal Root Ganglia and the Axon Initial Segment (AIS)

**Figure 2:**
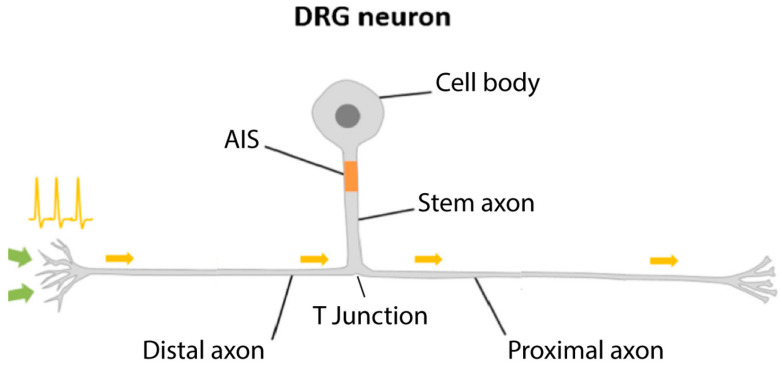
Diagram showing axon initial segment (AIS) essential for propagation, integration and modulation of the action potential at the bifurcation of the axon into the proximal and distal branches. Green arrows indicate the site of activation of the sensory neuron in peripheral tissues. Orange arrows indicate the afferent direction of action potential transmission to the spinal cord along the neuron. Adapted with permission from Ref. [6], Progress in Neurobiology. p 97. Elsevier.

During human development the dorsal root ganglion (DRG) neuronal cell body initially develops a pseudo-unipolar axon with prominent nucleated cell bodies, axonal stem and long axonal processes. Interestingly this was first described by y Cajal [9] and occurs in unmyelinated C, Aδ and Aβ myelinated neurons. Considering the complex structure and function of DRG neuronal cells, it is more relevant to refer to such neurons as C neurons or Aα and Aβ neurons rather than the commonly used term “fiber”. This nomenclature will therefore be used throughout. Of note is the axon initial segment (AIS), a highly specialized compartment in somatosensory neurons [6] which describes the initial area of the axon arising from the cell body and the ‘stem axon’ with a specialized T junction where the axon bifurcates into distal (to the skin and muscles) and proximal (to the spinal cord) axons. Recent studies of the active role of the stem axon and the axon initial segment (AIS) have emphasized the centrality of the cytoskeleton in neuronal structure and function [7]. This zone is now recognized as playing a major role in the propagation, integration and modulation of the action potential [6,7,10,11,12]. The development and specialization of the neuronal cell body and its axonal specialization is dependent on the associated Schwann cells and satellite cells [6] both of neural crest origin. It is important to note that although the distal axons in humans such as those innervating the legs can be up to a meter long, or as in blue whales, 30 m [13], the AIS maintains a central role on the function of the neuron “setting” the excitability of the whole neuron even at these distances (Figure 2).

Recent studies of the AIS junction report that axonal microtubule organization varies between proximal and distal axons with the proximal axon having a higher density of microtubules than the distal axon. The AIS is essential for two major functions: regulating the transmission of bio-electrical signals from the neuron’s cell body to its axon by initiating action potentials and maintaining neuron polarity [14] and, critically, controlling fast and slow transport as well as the distribution of proteins and organelles [15]. It is also of particular interest in understanding the role of mitochondrial transport along the intra-axonal cytoskeleton of relevance to PBMt effectiveness. The stem axon, AIS and the axonal bifurcation site are also the central areas for triphosphorylation of mitochondria which is reliant primarily on neuronal cell body metabolism. Interestingly and puzzlingly, axons have relatively fewer ribosomes and other cytoplasmic components in contrast to their neuronal cell bodies. They are dependent on mitochondrial phosphorylation and the delivery of ATP-rich mitochondria to the proximal and distal axons carried by molecular motors and kinesins on the cytoskeleton to maintain both the structure and function of the neuron.

### 3.2. Fast Axonal Flow (FAF)

Within the AIS, the cytoskeleton undergoes rapid and extensive structural and functional changes in response to neuronal activity levels and is the key regulator responsible for the unique neuronal morphology both within the ganglia and along the AIS and T junction. The uniquely organized intra-axonal microtubules of the cytoskeleton which are composed of α and β tubulin dimers undergo rapid de- and repolarization with a half-life of 10 min [16]. They extend distally from the nucleus in the cell body to skin, receptors, and muscles, and proximally, over a much shorter distance, to the synapse with the second-order CNS neurons within the relevant spinal cord segment. Microtubules provide the infrastructure for FAF, both anterograde and retrograde, along the length of each axon from the cell body to the periphery or CNS synapse. Triphosphorylation of mitochondria occurs within the AIS, and the ATP-rich mitochondria are carried by kinesin molecular motor proteins ‘walking’ anterogradely along microtubules [17].

**Figure 3:**
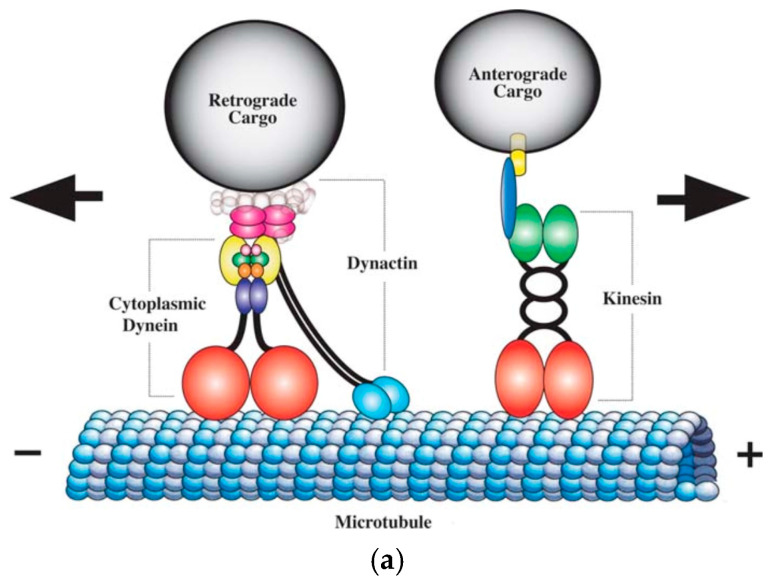

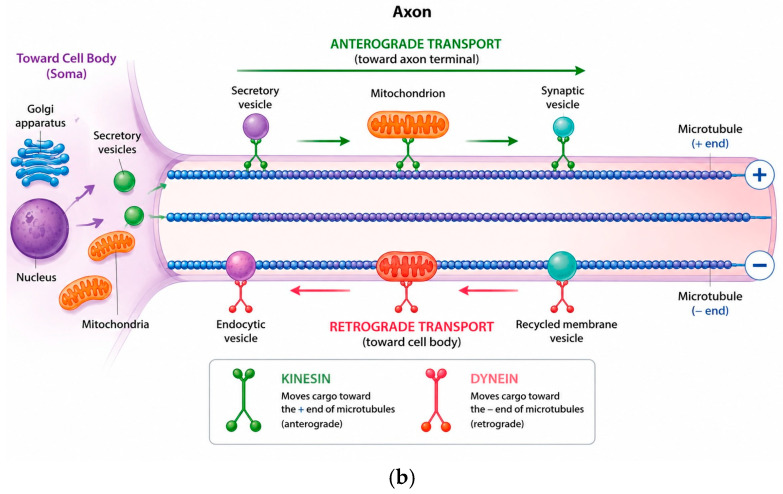
(**a**) Diagram of indicative microtubules within axons of peripheral neurons and their association with molecular motor proteins kinesin and dynein which are responsible for organelle transport particularly of mitochondria. Permission under Creative Commons Attribution License from Ref. [17]. (**b**) Diagram of an axon of a peripheral nervous system neuron, showing microtubules transporting mitochondria and other cargo anteriorly and posteriorly dependent on kinesin or dynein motor proteins. AI-generated image using ChatGPT (Version 1.2026.160).

Dephosphorylated mitochondria and other organelles including neuropeptides are transported retrogradely back to the cell body by dynein molecular motor protein [18]. FAF is important not only for mitochondrial movement along the axon but also for the translocation of other signaling molecules from peripheral terminals to the cell body in the DRG, such as nerve growth factor (NGF) and brain-derived nerve growth factor (BDNF) associated with pain modulation and nerve and tissue repair [19] (Figure 3a,b).

### 3.3. The Schwann Cell

**Figure 4:**
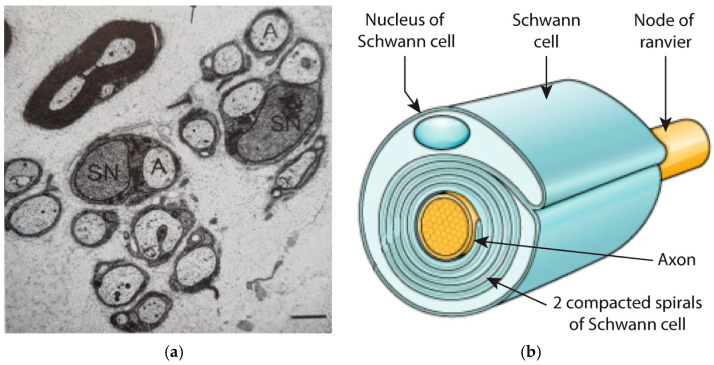
(**a**) Electron micrograph of human nerve showing cross section of nerve showing unmyelinated C-axons ensheathed by Schwann cells. SN: Schwann cell nucleus; A: axon, bar: 1 µm. Reprinted with permission from Ref [20] Elsevier License Number: 6276701457774 © 2005 Elsevier. (**b**) Myelinated axons ensheathed by spirals of compacted Schwann cell membrane Aδ and Aβ neurons. Adapted from https://commons.wikimedia.org/wiki/File:Periferal_nerve_myelination.jpg (accessed on 13 June 2026) under the Creative Commons Attribution Share Alike 3.0 CC BY-SA 3.0. Modified with permission under the terms of the license.

PNS and CNS axons are referred to as myelinated or unmyelinated based on examination at the light microscope level. Unmyelinated axons are ensheathed by uncompacted Schwann cells whereas myelinated axons are characterized by spiraling compacted ensheathment of individual Schwann cells along each axonal length [20] (Figure 4a,b).

Myelinated axons of Að and Aβ neurons are further characterized by nodes of Ranvier which develop where each Schwann cell abuts the other. The nodal and paranodal architecture is a complex arrangement of axon and Schwann cell membranes [21] and is essential for normal nerve conduction as its configuration affects nerve function, maintenance and response to injury or damage. In contrast, C neurons with their smaller diameter axons are unmyelinated but also ensheathed either individually or in groups within grooves of the Schwann cells. The number of Schwann cell spirals around each internode determines nerve cells as Aβ or Að neurons. The speed of conduction along myelinated axons depends on the number of compacted spirals of Schwann cell ensheathment with thinly myelinated Aβ neurons characterized by 100 or more spirals of compacted Schwann cells and conduction velocities of 30–120 ms. Myelinated Aδ neurons have a conduction velocity of 4–30 ms. These neurons respond to light, touch and pressure while unmyelinated C neurons lying within Schwann cell grooves with conduction velocities of <2.5 ms convey noxious sensation and thermoreception.

Within the relevant spinal cord segments, proximal axons of the first-order neurons synapse with dendrites of CNS second-order afferent neurons within the dorsal horn of the spinal cord with their axons ascending to relevant neurons within the cortical centers of the brain [22]. Peripheral nerve axons unlike those of the CNS do not form dendrites despite this being frequently stated in peripheral nerve literature and diagrams. However, their proximal axonal endings synapsing within the dorsal horn are MAP2-positive, characteristic of CNS axonal dendrites [23]. Again, of equal importance to neuronal function and generally overlooked is the recognition of the role of glial cells including not only SC but also the nerve cell body ensheathing satellite cells. Both are of neural crest origin and essential for normal nervous [15] system function but also in repair with a proportion of SC precursors also able to transition to ‘neuronal’ fibroblasts. These remain within the nerve, providing extracellular matrix molecules, mainly collagen, that occupy the space among different axonal fibers [24]. Indeed, recent studies have discovered new functions for SC precursors as a class of multipotent progenitors, which can generate several different cell types [15,25,26]. This configuration too is essential for normal function and in development, as well as maintenance and repair of the neuron. To complicate matters further, Abdo et al. have reported nociceptive SC which signal neuronal nociceptors in the upper dermal/epidermal region [27] increasing the evidence of their supporting role in pain. The complexity and importance of the Schwann cell in PNS function have been described in detail in a recent review [28].

## 4. Photobiomodulation Therapy (PBMt)

**Figure 5:**
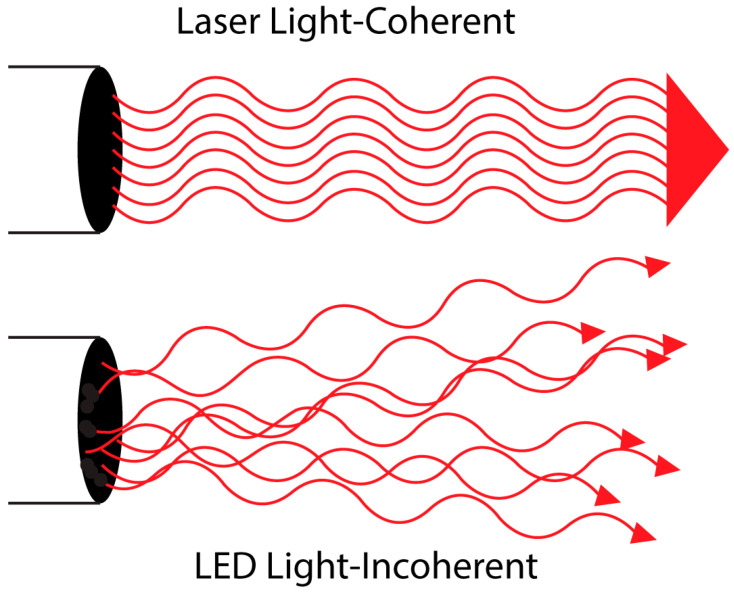
Coherent (laser) versus incoherent (light-emitting diode) light. Image created by Dr. Adahir Labrador-Garrido used with permission.

PBMt describes the interaction between photons and cells at sub-ablative fluences to modulate cell physiology to achieve therapeutic effects, and specifically for the focus of this review, pain modulation. PBM is the MESH term which now replaces older terms including low-level laser therapy (LLLT) or low-power laser therapy (LPLT) to include other light sources such as light-emitting diodes (LEDs) [29]. There are significant differences between laser and light emitting diodes (LEDs) which can alter therapeutic outcomes (Figure 5).

Coherence is one of the unique properties of laser radiation [30]. Whether or not coherence has therapeutic advantages over non-coherent light sources such as LED has been the subject of discussion among therapists and physicists [31]. Theoretical computations indicate that lasers diffuse and scatter very differently from LEDs within biological tissues.

**Figure 6:**
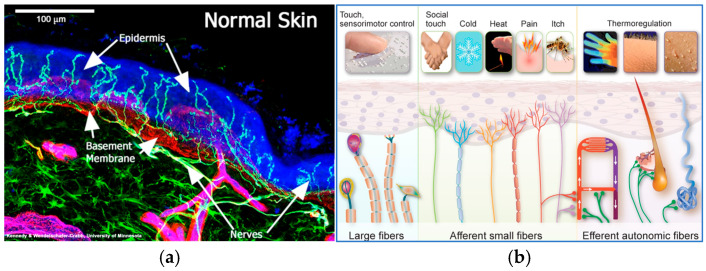
(**a**) Normal human epidermal and papillary dermis innervation. Nerve fiber (green and aqua) course in bundles through the dermis and branch to form the subepidermal neural plexus from where they penetrate the epidermal–dermal basement membrane to enter the epidermis. Reprinted with permission from Ref. [33] Saunders/Elsevier; 2005. Copyright © 2005. (**b**) Schematic view of skin innervation and functions of afferent and efferent nerve fibers. Reprinted with permission from Ref. [34], under Creative Commons CC-BY license 2024 © The Authors published by Elsevier.

Coherence may influence light distribution in tissue, and it can be demonstrated that lasers produce light speckles or pockets of intense light within tissues. Lasers therefore have the potential for a greater depth of penetration than LEDs. In viable, in vivo biological tissue, penetration is an important therapeutic factor in delivering photons to target cells or molecular structures which are surrounded and regulated by a complex infrastructure. In contrast, in monolayer cell cultures, penetration is not a prerequisite for delivery of an appropriate dose to achieve a biological effect. Indeed, in vitro, coherent and non-coherent light with the same parameters (i.e., wavelength, dose, intensity) produce the same biological effects. [32]. From a clinical perspective, the use of LED can be effective in treating conditions such as diabetic neuropathy which involves damaged C neurons which lie only a few microns under the keratin in skin and does not require significant penetration as can be seen in the following images [33,34] (Figure 6a,b).

### PBM “Dose”

PBM “dose” is complex and of great importance in understanding light/tissue interactions central to the efficacy of PBM [35]. Variables critical to “dose” and application include wavelength (λ), power output (W), time of exposure (seconds or minutes), “spot size” and mode (pulsed or continuous wave). These parameters allow calculation of total number of Joules (J), energy density or fluence (ED: J/cm^2^), and power density (PD) or irradiance (W/cm^2^). The World Association of Photobiomodulation (Laser) Therapy (WALT) now renamed has developed guidelines for some clinical conditions in order to standardize “dose” for clinicians [36]. Inconsistent reporting of parameters across studies is a point of weakness in PBM research with inappropriate doses contributing to negative studies [37]. Many publications have stressed the importance of accurately reporting parameters for reproducibility and this remains an ongoing challenge for researchers in this field [38].

## 5. PBM and Somatosensory Neurons

### 5.1. Varicosity Formation in Primary DRG Neurons in Culture

Fundamental to defining a neurophysiological mechanism for pain relief is the exploration of the role of the cytoskeleton. Few studies have investigated such PBM-induced structural changes. Chen et al. was the first to describe “beading” or varicosity formation following 830 nm, 20 mW, continuous-wave (cw) laser irradiation (LI) in isolated mouse DRG neurons [39]. Varicosity formation is significant as it indicates disruption of microtubule integrity and FAF, affecting the function of the whole neuron. Importantly in this study, LI decreased the number of substance P-positive neurons and calcitonin gene-related peptide (CRGP) staining neurons, characteristic of nociceptors indicating selective effects on nociceptors, relevant for the mechanism of pain-relieving effects.

**Figure 7:**
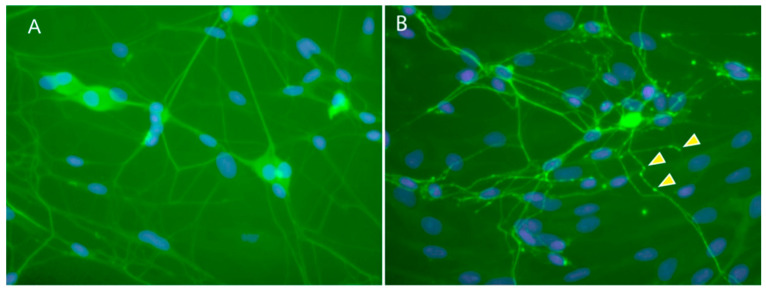
(**A**) Representative photomicrograph of control neonatal neuron cultures stained with anti-tubulin antibodies showing nerve cell bodies and smooth axonal arrays. (**B**) Representative photomicrograph of laser-irradiated neonatal neuron cultures (830 nm, cw, 30 s) showing varicosity formation along the axon (yellow arrows). Reprinted with permission from Ref. [40], Copyright © 2007 Wiley Periodicals, Inc.

Our group examined cytoskeletal changes following 650 nm, 808 nm and 830 LI in primary cultures of neonatal rat DRG neurons. Live imaging and immunohistochemical changes in axons following 830 nm LI demonstrated reversible varicosity formation representing depolymerization of microtubule subunits, α and β tubulin dimers [40]. Observation over 24 h showed reversal of “varicosities” (Figure 7A,B).

### 5.2. Microtubule Depolymerization and Mitochondrial Membrane Potential (MMP)

**Figure 8:**
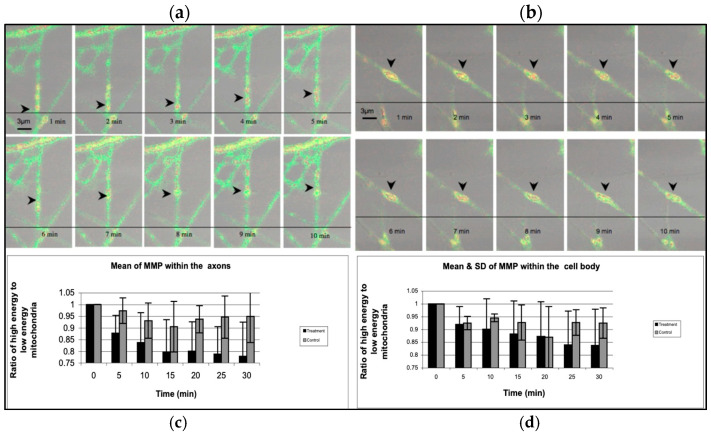
(**a**) Real-time confocal JC-1-stained axons after 30 s of 830 nm, cw, LI showing static varicosities (black arrows) indicative of cessation of FAF at 10 min within the axons. (**b**) Real-time confocal images of JC-1-stained, sham-irradiated control axons showing normal FAF movement of mitochondria (black arrows). (**c**) Histogram showing decrease in MMP in axons following LI. (**d**) Histogram showing decrease in MMP in cell bodies, both after 30 s, 830 nm, cw laser irradiation. Reprinted with permission from Ref. [40], Copyright © 2007 Wiley Periodicals, Inc.

Using real-time confocal microscopy, (Leica TCS SP2 Leica Microsystem Heidelberg GMBH) our group also reported cessation of movement of mitochondria along the microtubules with mitochondrial clusters accumulating in varicosities over 10 min observation of JC-1-stained axons following LI (830 nm, cw, 30 s). Simultaneously the mitochondrial membrane potential (MMP) declined reflecting a depletion of ATP (Figure 8a–d).

Using similar methodology with primary cultures of rat DRG neurons, Holanda et al. also identified varicosity formation primarily in small-diameter neurons (<30 µm) following 808 nm, 100 mW, cw LI [41]. Zupin et al. used adult rat primary DRG cultures and demonstrated increased MMP following 800 nm LI, in contrast to our findings and those of Holanda et al. where 970 nm did not change MMP [42]. Interestingly, reactive oxygen species were significantly increased at 15 min post-LI with both wavelengths and ATP levels were reduced by both wavelengths.

**Figure 9:**
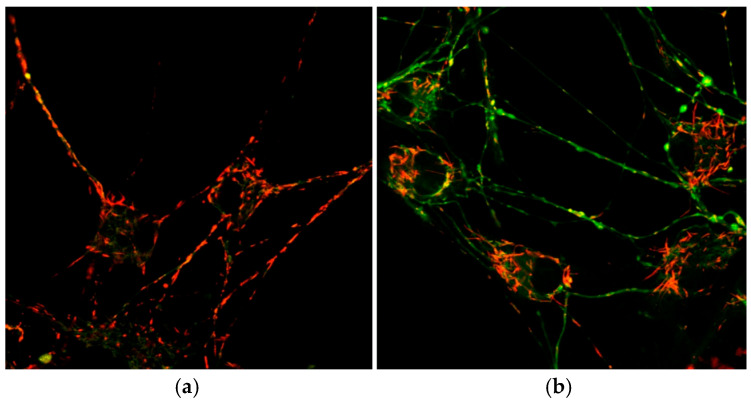
Confocal images of rat dorsal root ganglia in vitro stained with JC-1, a mitochondrial vital stain, showing axons and cell bodies, (**a**) prior to laser irradiation, demonstrating red staining indicating phosphorylated mitochondria within axons and neuronal cell bodies and (**b**) 1 min after 650 nm for 30 s showing pronounced decreased mitochondrial membrane potentials (green) in axons. Reprinted with author permission from Ref. [43] © 2012.

The previous studies have used near-infrared wavelengths, 780–1064 nm, most commonly used in the treatment of pain. For the first time our group also demonstrated that red, 650 nm LI also caused reversible varicosity formation and functional changes in microtubules as seen with 830 nm LI in the same cultured rat neonatal DRG neuron model. Importantly and extending the understanding of the translational perspective of these findings, immunohistochemistry demonstrated TRPV-1 fluorescence in the small- and medium-diameter neurons, nociceptors, which were selectively affected. The effects of a single irradiation of 650 nm LI on axonal MMP (ψmmp) caused the formation of axonal varicosities. There was no difference in the number of varicosities and mitochondrial clusters which were still present 24 h post-LI for all exposure durations. MMP was also measured showing no significant change in axonal MMP in axons irradiated for 15 s compared to sham neurons; however, axonal MMP in neurons irradiated for 30 s was significantly decreased at 8 min (*p* < 0.05, *p* value = 0.04). Neurons irradiated for 60 s demonstrated significantly lower MMP values throughout the ten-minute imaging period [43] (Figure 9a,b). A reduction in FAF following 30 s and 60 s 650nm LI was also seen, consistent with decrease in MMP in axons (Table 1).

### 5.3. Disruption to Na^2+^K^+^ATPase—Depolarization Blockade

The accumulation of ATP-depleted mitochondria in varicosities, disruption of FAF and MMP decline, which are reversible, are reflective of a significant change in the function of the whole neuron. Microtubule depolymerization initiates a cascade of disruption to nerve function, particularly the supply of ATP to ATPase-requiring enzymes such as Na^+^K^+^ATPase, a voltage-gated sodium channel setting the baseline for generation of action potentials. This results in depolarization blockade. Kudoh et al. using a rat saphenous nerve model demonstrated a biphasic response to LI (830 nm, cw, 60 mW laser) in Na^2+^K^+^ATPase activity with an increase at lower doses of LI and suppression at higher doses [44]. The function of many other ATP-dependent enzymes in neurons would also potentially be disrupted by microtubule depolymerization and reduced ATP availability [45].

Supporting these findings Miura and Kawatani used a patch-clamp technique in rat nodose ganglion cultures and showed that 830 nm LI at varying power outputs caused dose-dependent depolarization in 67% of small-diameter (<32.7 µm) neurons [46]. In a parallel study, using a voltage clamp technique, a sodium channel blocker, tetrodotoxin (TTX), abolished laser-induced depolarization, suggesting that LI acts on Na^+^ channels in primary sensory neurons to cause depolarization. Similar depolarization of Na(v)1.8 channels was observed using low-power CO_2_-laser using the whole-cell patch-clamp membrane of cultured neonatal rat DRG neurons [47]. Ouabain, known to block both functions of Na^+^K^+^ATPase, eliminated IR irradiation effects. The authors proposed that CO_2_-laser decreased voltage sensitivity of Na(v)1.8 channels with the potential for a nociceptive effect. The selective PBM inhibition of sodium channel activity in nociceptors contributes to further understanding its mechanism of action in pain relief [48].

### 5.4. The Cytoskeleton and Pharmacological Agents

This putative mechanism of PBM aligns with the mechanism of action of certain drugs which disrupt the integrity of the microtubules, for example, colchicine, used in the treatment of gout or *Vinca Alkaloids* in cancer. Similarly, the mechanism of action of local anesthetic agents arises in part from disruption of cellular cytoskeletal systems with lidocaine specifically impairing microtubule polymerization [49,50,51].

## 6. PBM Blocks Action Potentials—Electrophysiology

To extend the translational aspect of this review from individual neurons to whole nerve models, we report a series of human and animal studies, in vivo and in vitro, which demonstrate inhibition of action potential amplitude and increased latency. A systematic review of the literature was undertaken up to 2011 resulting in a publication which reported on electrophysiological studies of PBM in wavelengths up to 980 nm [52]. Additional studies, including those using 1064 nm laser, published since then have been included in this review.

### 6.1. Human Electrophysiological Studies

In human subjects, reduction of action potential amplitude and/or increase in latency have been described in studies following transcutaneous application of LI over the median nerve [53,54,55,56,57], superficial radial nerve [58,59,60,61,62], sural nerve [63,64], including an LED study of sural nerve [65] and in one study of LI on trigeminal nerve [66]. Studies of PBM on conduction in the ulnar nerve also show reductions in action potential amplitude and/or increased latency [67]. LED appears to be less effective in slowing conduction than laser. Overall, these studies show evidence that multiple IR wavelengths of transcutaneous LI can increase nerve latency and reduce action potential amplitude in a dose-dependent manner in human nerves. Studies using very-low-dose red laser generally show no effect, and pulsation of IR wavelengths, which reduces total incident laser dose, also limits the effect on action potentials in some studies (see Supplementary Materials A).

### 6.2. PBM-Induced Action Blockade in Animal Studies

Transcutaneously delivered or directly applied LI to exposed nerve in animal experiments demonstrates similar reduction in action potentials as in human subjects. Our group demonstrated that transcutaneous 650 nm or 808 nm LI delivered along the course of the sciatic nerve in an in vivo model increased latency and decreased amplitude of somatosensory evoked potentials (SSEPs) and compound muscle action potentials (CMAPs) within 10 min of application. In particular, 650 nm LI showed a small but significant effect which persisted at 24 h but returned to normal by 48 h [68]. Other studies using different nerve models show similar findings. For details see Supplementary Materials B.

### 6.3. PBM-Induced Blockade of Noxiously Generated Action Potentials

Although the reduction in action potential amplitude and increased latency in both animal and human studies following LI is putative evidence for pain modulation, from a translational perspective, PBM-induced reduction in noxiously generated action potentials is more definitive evidence for a neural mechanism for pain modulation. In several in vivo studies, LI inhibited pro-inflammatory and noxiously evoked potentials elicited by mechanical [69], thermal [70,71,72] or chemical stimulation, such as formalin, turpentine, or bradykinin injection [73,74,75,76]. Studies of LI electrically evoked compound action potentials (CAPs) in several animal models were evaluated using various methodologies showing inhibition of action potential amplitude [46,74,77,78,79,80], SSEPs [81]. In a series of experiments using Nd:YAG LI (1064 nm), Wesselmann et al. demonstrated cytoskeletal disruption and decreased action potential amplitudes of in vitro rat nerve following [82,83,84,85,86,87,88]. Many of these studies can be regarded as legacy studies and details of PBM parameters, in particular, are not reported fully; however, they do provide a strong body of evidence for specific, PBM anti-nociceptive effects and a basis for further research. For details see Supplementary Materials C.

### 6.4. PBM on Peripheral Nerve Reduces Action Potential at Spinal Cord Level

Observations in a series of animal models show that PBM can inhibit action potentials generated in peripheral nerves and recorded at DRG or spinal cord laminae give further insight into the mechanism for disruption of upstream “pain” signals at second-order neurons. Kono et al. identified reduction in spinal cord evoked potentials in cats following LI to applied to the sural nerve [77]. Tsuchiya et al. demonstrated blockade of APs at the dorsal root initiated by multiple forms of noxious stimuli delivered to rat paw [79]. Uta et al. showed reduced neuronal firing in lamina II of the spinal dorsal horn of rat evoked by mechanical stimulation with von Frey filaments following 808 nm LI to the exposed sciatic nerve [89]. The evoked potentials from nociceptors were inhibited from 5 min after LI and persisted for 3 h. Histopathological evaluation revealed no damage to the sciatic nerve due to LI. Shimoyama et al. demonstrated that prolonged (30 min), transcutaneous application of LI to the hind paw of anesthetized rats attenuated formalin-induced action potentials of the dorsal horn neurons at L1–L3 [90]. Buzza et al. 2024 have more recently also demonstrated selective C neuron inhibition using 808 nm, 60 mW, and 4 min application in an exposed sciatic nerve model [91].

In another experimental model, horseradish peroxidase (HRP), a cell marker used to label sensory neurons and a marker of axonal transport, was applied to the proximal end of severed rat tibial nerve, which had been laser-irradiated seven days earlier [86]. A selective decrease in the number of small HRP-labeled DRG cells in nerves exposed to LI compared with no change in larger sensory neurons or motor neurons strongly suggests that 1064 nm LI selectively disrupted retrograde flow in Aδ and C nociceptors [92]. Wesselmann also demonstrated reduction in action potentials in small sensory nerves [84].

### 6.5. PBM to Peripheral Nerve Mitigates Noxiously Evoked Pain Behaviors

Mitigation of pain behavior following LI to peripheral nerves was observed in several animal experiments. Holanda et al. showed significant improvement in cold and heat allodynia in LI-treated rats tested with von Frey hairs compared with control in an in vivo spared nerve injury (SNI) model of neuropathic pain. Mechanical allodynia was unchanged [41]. Zupin et al. in a mouse model showed that transcutaneous 800 and 970 LI reduced pain behaviors following capsaicin injection to the paw [42]. Buzza et al. demonstrated that PBM at 808 nm delivered directly to the exposed sural nerve reduced pain sensitivity to noxious heat and mechanical stimuli in an in vivo sural nerve, SNI model [91]. They extended the research to the examine response in the sciatic nerve, a mixed nerve, and demonstrated significant reduction in Aδ and C-neuron-mediated sensitivity to mechanical (pin prick) and chemical (capsaicin) noxious stimuli. Importantly, there was no change in Aα and Aβ motor nerve activity [93].

### 6.6. Animal Studies Relevant to Dental Application

Animal studies specific to dental application relate particularly to the observation that LI over the face, the receptive field for the trigeminal nerve, reduced axon potentials in the trigeminal nerve and nucleus. Wakabayashi [73] et al. showed suppression of late discharges from C neurons, by 120 s 830 nm, 350 mW, cw LI applied to an incisor following electrically evoked responses in tooth pulp recorded in ipsilateral TSC neurons in anesthetized Wistar rats. Early discharges from Aδ afferents were not affected. The authors concluded that low-power LI suppressed action potentials by depolarization blockade of C neurons.

Maeda demonstrated that 830 nm, 60 mW LI to the skin of the face innervated by the maxillary branch of the trigeminal nerve blocked mitochondrial density increases which occurred following bradykinin injection to facial skin [94]. This too is relevant for conditions such as trigeminal and post-herpetic neuralgia of the face.

In an in vitro model, Orchardson et al. showed that application of pulsed Nd:YAG dental laser (dLase 300) applied in static contact with the surface of a root segment of a tooth with a rat spinal nerve lying within a prepared pulp canal irreversibly abolished CAP in the nerve [95]. This study demonstrated that LI caused a dose-dependent block of action potential conduction in nerve fibers in the underlying pulp chamber. In a second study by the same author, Nd:YAG laser had a variable dose-dependent effect on intradental nerve response to mechanical stimulation of exposed dentine stimulation of canine teeth of anesthetized ferrets; 0.6–1.5 W could either enhance or suppress intradental nerve responses, and lasing at more than or equal to 2.0 W or repeated lasing at lower intensities depressed intradental nerve responses [69]. Both the Orchardson and Wakabayahsi studies provided a basis for the later clinically based studies of Chan et al. described below.

## 7. Clinically Relevant DRG Studies

### 7.1. Human DRG Study—In Vivo

The importance of the AIS within the DRG neuron, which plays a major role in the propagation, integration and modulation of the action potential, has been emphasized previously. It therefore presents as a potential target for PBMt as a logical research step. Testing the hypothesis of its central role in PBM-related pain modulation, Holanda et al. targeted DRG in a novel clinical trial of patients with low back pain [96]. Low back pain score was assessed by the Visual Analog Scale (VAS) and Pain Relief Scale (PRS) pre- and post-procedure. Laser was delivered via a fiber-optic device, (DMC Equipamentos Ltda, São Paulo, Brazil), to the DRG at the second lumbar vertebral level in patients with back pain (parameters—Table 2). Controls were treated with lidocaine or radiofrequency ablation of L2 nerve roots, both standard interventional procedures in pain medicine (Table 2).

In this study, a single PBM session resulted in significant pain relief for all participants, with more than 50% experiencing complete pain alleviation on day 1, and 70% of patients reporting more than 50% pain relief at 1 month. These results support the clinical validity and feasibility of targeting the DRG; however, this technique requires specialist training and hospital facilities. To date, no other study has replicated this clinical trial. Jenkins et al. describe this direct application of PBM to DRG as transient small-fiber neural inhibition (tSNIP), which is consistent with specific C neuron inhibition [97]. The rationale for defining tSNIP through its specific therapeutic effect on nociceptors contrasts with general PBM applications.

### 7.2. Animal DRG Studies—In Vivo

Supporting the importance of DRG as a therapeutic target are two animal experiments [98,99]. Each reported significant but different changes. Chen et al. targeted DRG neurons in the fourth and fifth lumbar intervertebral foramina in a rat chronic compression model (CCD), a validated model used to study neural responses and pain-related behaviors, using transcutaneous laser (808 nm, 300 mW, cw) for 8 consecutive days following CCD surgery. Messenger RNA (mRNA) expression and expression of pro-inflammatory cytokines, TNF-α and IL-1β, were increased in the CCD control group, but were significantly reduced by LI. In addition, LI significantly decreased hyperalgesic response to pain and heat stimuli in the CCD group compared with controls.

De Sousa et al. in a mouse model demonstrated a 3-fold increase in pain threshold using von Frey filaments in the right hind paw of mice following LI (810 nm, cw) to the lower back overlying the DRG [99]. The optimal effect occurred 2–3 h post-PBM which disappeared by 24 h. PBM to anatomical sites sharing the same spinal pathways such as the head, neck and ipsilateral (right) paw also produced an analgesic effect but the optimal effect was achieved with LI applied to the DRG. LI applied to areas outside the nerve root distribution had no effect. Seven daily irradiations showed no development of tolerance.

These studies provide the clinical plausibility of targeting the DRG as part of clinical protocols for the treatment of pain and should be a focus for future research.

## 8. PBM in Clinical Studies

### 8.1. Clinical Studies of PBM in Dentistry

Use of PBM routinely in dentistry is increasingly accepted world-wide based on a three-decade history of clinical research, starting in the early 1970s [100]. An example of a more recent study is that by Chan et al. in a double-blind, randomized, clinical trial which tested the effectiveness of pulsed Nd:YAG laser (1064 nm) induction of pulpal analgesia compared with 5% EMLA anesthetic cream [101]. Analgesia was tested by an Electric Pulp Tester (EPT) and the cutting of a standardized cavity. This trial confirmed that pulsed Nd:YAG laser effectively induced pulpal analgesia and was associated with reduction in intradental nerve sensitivity. This study illustrates the potential of PBMT in painless, non-invasive dentistry. Subsequent clinical trials show the effectiveness of PBM in analgesia and anesthesia currently used in routine dental and orthodontic practice [4,102,103,104,105]. Substantial evidence from systematic reviews in dentistry also includes temporomandibular joint disorders (TMDs) [106,107,108], endodontics [109], burning mouth syndrome [110,111], orthodontic movement pain [112], laser-induced analgesia [113], post-operative pain [114,115], and oral mucositis [116,117]. WALT has recently published a position paper on the management of orofacial neuropathic pain to guide optimal treatment parameters [118]. Refining research into optimal parameters for PBM applications in dentistry will continue to improve outcomes in difficult-to-treat clinical conditions. See details in Supplementary Materials D.

### 8.2. Clinical Studies of PBM in Medical Conditions

From the earliest days of clinical application of PBM, LI and more latterly LED have been applied transcutaneously to achieve therapeutic effects in both nociceptive and neuropathic pain conditions. There are now several significant systematic reviews and randomized controlled trials of PBM for multiple painful medical conditions, including neck pain [119], knee osteoarthritis [120], diabetic peripheral neuropathy [121], shoulder tendinopathy [122,123], neuropathic pain [124], chemotherapy-related neuropathy [125] and musculoskeletal pain [126]. Other clinical trials and reviews have reported favorable outcomes in PBM delivered transcutaneously in back pain [127,128], trigeminal neuralgia [129], post-herpetic neuralgia [130], fibromyalgia [131], and crystalline arthropathies [132,133]. See details in Supplementary Materials E.

## 9. Limitations to the Review

In developing a neurophysiological hypothesis for pain modulation, we have focused explicitly on evidence from studies of direct PBM effects on somatosensory nerves and the PNS. This addresses the PBM applications most specific to neural conditions such as neuropathic pain conditions, myofascial pain syndrome, analgesia and anesthesia. The complex cascade of intra- and extracellular signaling initiated by photon absorption by cytochrome c oxidase in mitochondria in multiple compact cell types, such as fibroblasts, macrophages and neutrophils, has been well described and underpins many other diverse applications of PBMt such as wound healing and sports performance [134].

## 10. Future Perspectives

This review provides an overview of neurophysiological mechanisms of pain modulation. In any clinical application, however, a reductionist perspective of PBM effectiveness based on responses of laser or LED on cells, isolated tissues or neurophysiology will never replicate or fully elucidate the complex biochemical and physiological response in any one individual with a unique genome. The forty-decade-long history of clinical application attests to the clinical acumen of early researchers who achieved positive results with limited understanding of laser–tissue interactions. Sophisticated systems-based research approaches in clinically based research, with the assistance of AI, will be necessary to fully unravel the complexity of PBM in clinical applications. In relation to neurophysiological mechanisms, further research should continue to explore the effects on the PNS, in particular, an examination of Schwann cell–neuron interactions which are critical to nerve function and repair, and clinical applications to target the DRG. This review will provide important therapeutic directions for nerve injuries and diseases including the possibility of spinal cord repair.

## 11. Conclusions

This review illustrates the convergence of findings across diverse experimental methodologies. These, in turn, correspond with clinical observations of analgesia and altered sensory function. While individual mechanisms remain incompletely understood and further research is warranted, the collective body of evidence demonstrates biological plausibility and internal consistency across multiple levels of investigation. They reflect a systems-level neurophysiological basis for pain modulation and support the hypothesis that PBM modulates the somatosensory nervous system as an independent mechanism for pain modulation in medicine and dentistry and provide a platform for further research.

## Figures and Tables

**Table 1:** Reduction in speed of FAF immediately following 30 s and 60 s LI and after 24 h.

Wavelength 650 nm + Time	ED	FAF Time 0©	FAF @ 24 h	FAF Control	*p* Value
Experiment 1: 30 s	10.2 J/cm^2^	0.17 μm/s	0.27 μm/s	(average) (0.5–2 μm/s)	*p* < 0.011
Experiment 2: 60 s	20.4 J/cm^2^	0.07 μm/s	0.11 μm/s	(average) 0.50 μm/s (0.5–2 μm/s)	*p* < 0.0001

**Table 2:** Parameters of laser device in clinical trial by Holanda et al.

Wavelength	Mode	Output Power	Total Energy	ED	PD	Spot Size	Device
808 nm	cw	100 mW	8.4 J	2800 J/cm^2^	35 W/cm^2^	0.003 cm^2^	Photon LaseIII DCM, Brazil

## Data Availability

No new data were created or analyzed in this study. Data sharing is not applicable to this article.

## Supplementary Materials

The following supporting information can be downloaded at https://www.mdpi.com/article/10.3390/cimb48070695/s1.

## Abbreviations

LI	Laser irradiation (light with the characteristics of laser used in experimental studies)
LED	Light-emitting diode (light with the characteristics of light-emitting diodes)
PBM	Photobiomodulation—the general term for the use of non-ablative light (either laser or LEDs) modulating biological processes
PBMt	The application of PBM in a therapeutic manner
IR	Infrared
DRG	Dorsal root ganglion/ia
AIS	Axon initial segment
MMP	Mitochondrial membrane potential
FAF	Fast axonal flow
LLLT	Low-level laser therapy
