# Supplementary material for: A Translational Review of Mechanisms of Effectiveness of Photobiomodulation on Somatosensory Neurons and the Peripheral Nervous System—From Molecular Mechanisms to Clinical Applications in Medicine and Dentistry
Source: Curr Issues Mol Biol. 2026 Jul 9;48(7):695. doi: 10.3390/cimb48070695 (PMC13409449; doi:10.3390/cimb48070695)
Supplement: Supplementary file 1 [file cimb-48-00695-s001.zip › Supplementary materials E med sys rev 22-6-26 .pdf]

Supplementary Materials E: Systematic Reviews of Photobiomodulation in Painful Medical Conditions.

Condition	Author	Year	Principal Finding
Trigeminal Neuralgia	Ibarra et al.; Taddeucci et al.	2021–2026	Evidence supports PBM for trigeminal neuralgia pain.
Neuropathic Pain	de Andrade et al.	2016	Potential benefit in neuropathic pain.
Diabetic Peripheral Neuropathy	Korada et al.	2023	Improved pain and nerve conduction.
Neck Pain	Chow et al.; Gross et al.	2009–2013	Consistent evidence of analgesic efficacy.
Low Back Pain	Tomazoni et al.; Chen et al.	2020–2022	Mixed findings.
Knee Osteoarthritis	Huang et al.; Stausholm et al.	2015–2019	Improved pain and function.
Fibromyalgia	Yeh et al.	2019	Reduced pain and symptom burden.
Shoulder Disorders	Haslerud et al.; de Lara Quagliotto et al.	2015–2025	Supported efficacy.
Tendinopathies	Bjordal et al.; Naterstad et al.	2008–2022	Improved pain and function.
Carpal Tunnel Syndrome	Franke et al.; Lauxen et al.	2018–2025	Mixed to positive evidence.
