# Supplementary material for: A Translational Review of Mechanisms of Effectiveness of Photobiomodulation on Somatosensory Neurons and the Peripheral Nervous System—From Molecular Mechanisms to Clinical Applications in Medicine and Dentistry
Source: Curr Issues Mol Biol. 2026 Jul 9;48(7):695. doi: 10.3390/cimb48070695 (PMC13409449; doi:10.3390/cimb48070695)
Supplement: Supplementary file 1 [file cimb-48-00695-s001.zip › Supplementary material A CIMB EV and human studies 22-6-26 .pdf]

Supplementary materials A: Human studies of LI suppression of action potential amplitude and/or latency.

Author and year	Nerve	λ (nm) and beam mode LI (unless stated otherwise)	Power and Rx parameters	Sites treated	Effect on conduction velocity	Effect on evoked action potential
Greathouse et al. 1985	Superficial radial n = 20	904, 73 Hz	Exp 1: 20 sec; Exp 2:120 sec	5 points at 1 mm above skin surface; observed at 1, 3, 5, 10, 15 min post-LI	Exp1: no change; Exp2: decreased $p<0.05$	Exp1: no change; Exp2: no change
Snyder-Mackler et al. 1985	Superficial radial n=20	632.8, cw 1 mW	? duration	6×1 cm points along course of nerve; 0.5 mm from skin	Decreased $p<0.05$	NR
Walker & Akhanjee 1985	Superficial radial and median n=10	632.5, 3.1 Hz 1 mW	Exp 1: 800 pulses; Exp 2: 4800 pulses	4 mm^2 area at each nerve; spot 4 mm^2	NR	Exp1: decreased; Exp2: decreased (prolonged exposure) by 20–90%; no response
Wu et al. 1987	Median n=9	632.5, 3.1 Hz 1 mW	Fiberoptic probe	4 mm^2 area	NS	NS
Snyder-Mackler & Bork 1988	Superficial radial n = 40	632.8, cw 1 mW	20 sec	6×1 cm^2 above skin; 2 mm points over nerve	Decreased by 14.2% $p<0.001$	NR
Basford et al. 1990	Radial & median	633; cw 1mW	? duration	6 points over nerves	NS	NS

	n = 40					
Baxter et al. 1990	Median n=27	830, cw 40 mW	ED 1.2 J/cm²	10 points over nerve in contact with skin	Decreased $p<0.05$	NR
Baxter et al. 1991	Median n=48	830, cw 40 mW	ED 1.2 J/cm²	10 points over nerve	Decreased (>1 h post-LLLT) $p<0.05$	NR
Baxter et al. 1991	Median n = 24	830, cw	40 mW; ED 1.2 J/cm²	10 points over nerve	Decreased post-LLLT	NR
Kramer & Sandrin 1993	Superficial Radial n = 40	Exp 1:780 cw Exp 2: 632.8 cw	Exp1:12 mW 15 sec; Exp2:10 mW 18 sec	6×1 cm²; 1 mm from surface	Exp 1 no change; Exp 2 decreased $p<0.03$	NR
Baxter et al. 1994	Median, n = 51	830 nm, cw	40 mW; 30 sec per point; 1.2 J per point; ED 9.6 J/cm²	Exp 1: 30 sec, 10 points along course of nerve. Exp 2: as above but 4 cm medial to nerve. Exp 3: 4 points along palm nerve.	Exp 1: dec. observation 1 hr ($p<0.05$). Exp 2: NS Exp 3: decreased ($p<0.05$).	NR
Lowe et al. 1994	Median, n = 80	830 nm, cw 30 mW	PD 300 mW/cm²; Exp 1: 1.5 J/5 sec; Exp 2: 3 J/10 sec; Exp 3 6 J/20 sec; Exp 4 9 J/30 sec; Exp 5 12 J/40 sec	10 points over nerve; 20 min observation at 2 min intervals; spot size 0.1 cm²	Exp 1–3 decreased ($p<0.05$); Exp 4–5 NS	NR
Lowe et al. 1995	Median, n = 90	820 nm pulsed; 12 Hz, 73 Hz and 5 kHz	46 mW av. power;	10 points over nerve in contact with skin	NS	NR

			ED 1.5 or 9.0 J/cm²; spot size 0.125 cm²; 368 mW/cm²; Exp 1: 3 0.18 J/4 sec; Exp 4–6: 1.1 J/24 sec			
Bartlett et al. 1999	Median, n=42 (4 groups)	830 nm, CW	90 mW; ED 33 J/cm²; PD 1 W/cm²; 33 sec × 4 = 12 J	Three multi-head applications; spot size 0.09 cm²; Exp1: LI at wrist single area; Exp2: LI at forearm, 2 areas treated separately	NS	No change at 0 and 10 min
Cambier et al. 2000	Sural, n=15	830 nm pulsed; 1–500 mW; 0–1500 Hz; PW 500 µs	Exp1: 400 mW, 3.8 sec/point, 1.5 J, ED 7.65 J/cm². Exp2: 140 mW, 5 sec, 1 J/point, ED 5.1 J/cm². Exp3: 30 mW, 16.6 sec/point, ED 2.55 J/cm², 0.5 J	6 points over nerve; spot size 0.196 cm²	Exp1 no change; Exp2 decreased ($p<0.05$); Exp3 no change	NR
Walsh et al. 2000	Superficial radial, n=32	820 nm, pulsed, 9.12 Hz & 73 Hz	46 mW, 0.125 cm²; ED 9.55 J/cm²; 368 mW/cm²; 1 J per point; Exp 1: 24 sec; Exp 2: 24 sec	3 points over nerve; 5, 10 and 15 min	NS	NR
Noble et al. 2001	Median n N=40	LED 890 nm; pulsed 270 Hz	PD 0.42 mW/cm2 Exp 1: ED: 1.7 J/ cm² Exp 2: 4.0 J/ cm²	Two arrays; 60 diodes; spot size 22.5 cm2 per array; along course of n	NR	Decreased $p< 0.028$

Nelson and Friedman 2000	Trigeminal nerve, maxillary branch, n=24	632.5 nm, pulsed, 50 Hz 1.7 mW	spot size 0.001 cm^2; PD 1.73 W/cm^2; ED 138.4 J/cm^2; 120 sec/point; 0.2 J	1 point at left maxillary 3rd molar apical area	Decreased to 65% at 10 min and 72% at 20 min ($p<0.001$)	NS
Hadian and Moghadam 2005	Sural, n=38	Exp 1–3: 670 nm CW; Exp 4–6: 780 nm CW	3 mW; Exp1: ED 0.5 J/cm^2, 5 J, 167 sec. Exp2: ED 1.5 J/cm^2, 15 J, 501 sec. Exp3: ED 2.5 J/cm^2, 25 J, 835 sec. Exp4: ED 0.5 J/cm^2, 0.075 J, 25 sec. Exp5: ED 1.5 J/cm^2, 0.150 J, 75 sec. Exp6: ED 2.5 J/cm^2, 0.325 J, 125 sec	Along course of nerve (number of points unspecified)	Exp 1–6 all decreased ($p<0.01$ to $p<0.001$)	Exp 1–6 all decreased ($p<0.01$ to $p<0.001$)
Savafavi et al. 2005	Superficial radial n = 40	780nm; 20mW	Exp 1:0.5J, Exp 2: 1.5J Exp 3: 2.5J	5 pts along nerve	Exp 1: NS Exp 2: $p < 0.001$ Exp 3: < 0.001	Exp 1: $p < 0.001$ Exp 2: $p < 0.001$ Exp 3: $p < 0.001$
Vinck et al. 2005	Sural, n = 64	LED; 160mW, cw	1.07 J/cm^2	Single point application in contact with skin	$p <0.003$	$p <0.006$
Telemeco et al. 2013	Superficial radial n = 32	LED cluster probe 32 x 808nm 4 x 660 LEDs	6J/cm^2 30s	2 areas overlying course of nerve	NS	NS
Suganthirababu et al. 2021	Ulnar n = 60	904nm	20s; ED: 4J/cm^2	One area at med epicondyle	$p < 0.021$	$p <0.01$

NR – not reported; NS – not significant.
