# Supplementary material for: A Translational Review of Mechanisms of Effectiveness of Photobiomodulation on Somatosensory Neurons and the Peripheral Nervous System—From Molecular Mechanisms to Clinical Applications in Medicine and Dentistry
Source: Curr Issues Mol Biol. 2026 Jul 9;48(7):695. doi: 10.3390/cimb48070695 (PMC13409449; doi:10.3390/cimb48070695)
Supplement: Supplementary file 1 [file cimb-48-00695-s001.zip › Supplementary material B animal cond 22-6-26 .pdf]

Supplementary Material B: Animal Studies of PBM Effects on Nerve **Conduction.**

**Study**	**Animal and nerve irradiated**	**λ (nm), beam mode, power**	**Site treated and duration of LI**	**Conduction velocity**	**Electrically evoked CAP or SSEP**	**Morphological or functional change**
Rochkind et al. 1986	Rat sciatic nerve (in vivo)	632.8 cw 16 mW	30 min transcutaneous	NR	Dose-dependent CAP changes	NR
Nissan et al. 1986	Rat sciatic nerve (in vivo)	632.8 cw 16 mW	30 min transcutaneous	NR	CAP amplitude changes	NR
Rochkind et al. 1988	Rat sciatic nerve (in vivo)	632.8 cw 16 mW	30 min transcutaneous	NR	43% increase after LI	NR
Kao et al. 1989	Dog sciatic nerve (in vitro)	632.8 IR	Exposed nerve	No change	Reversible SSEP decrease	NR
Arber et al. 1990	Rat sciatic nerve (in vitro)	633	Isolated segment	No change	NR	NR
Jarvis et al. 1990	Rabbit corneal nociceptors	632.5 4 mW	5 min	No change	No change	NR
Shimoyama et al. 1992	Rat superior cervical ganglia	632.8 5.5 mW	3–10 min	No change	Decreased responses	NR
Tsuchiya et al. 1993	Rat saphenous nerve	830 40 mW	30–180 sec	Fast component decreased	Evoked response decreased	NR

Kasai et al.<br>1996	Rabbit<br>sural nerve	632.8<br>1 mW	10 min	Aδ<br>conduction<br>↓ 9–19%	NR	NR
Ohno, T<br>1997	Rat sciatic<br>nerve	830<br>60 mW	Direct nerve<br>irradiation	Substance P<br>synthesis	Substance P at<br>DRGs	NR
Chow et al.<br>2006	Rat D<br>In vivo rat<br>sciatic<br>nerve	830<br>40 mW<br>650	30 s<br>At 4 pts along<br>nerve<br>Trans cut		Reduced<br>CMAP<br>and SSEP<br>amplitude	
