# Supplementary material for: A Translational Review of Mechanisms of Effectiveness of Photobiomodulation on Somatosensory Neurons and the Peripheral Nervous System—From Molecular Mechanisms to Clinical Applications in Medicine and Dentistry
Source: Curr Issues Mol Biol. 2026 Jul 9;48(7):695. doi: 10.3390/cimb48070695 (PMC13409449; doi:10.3390/cimb48070695)
Supplement: Supplementary file 1 [file cimb-48-00695-s001.zip › Supplementary Material C LI inhibits noxious stimulation 22-6-26.pdf]

Supplementary material C: Effects of laser irradiation, all wavelengths, on noxiously evoked stimuli.

**Author & Year**	**Animal**	**λ (nm)**	**Power & Beam Mode**	**Power and Rx Parameters**	**Noxious Stimulus**	**Neural Response**
Mezawa et al. 1988	Cat lingual nerve (in vivo)	904	2W; 3040Hz; pulse width 200ns	Exp 1: 60s<br>Exp 2: 180<br>Exp 3: 300s<br>Exp 4: 600s LI	Thermal—30s	AP amplitude<br>Exp 1: minimal change<br>Exp 2: inhibition<br>Exp 3: inhibition<br>Exp 4: inhibition
Maeda et al. 1989	Rat trigeminal nerve (max branch) (in vivo)	830	60mW, cw (transcut)	15s/pt PD: 1.9W/cm^2; spot size: 2mm^2 12pts at 2x/day for 7 days	Chemical—bradykinin	Inhibition of increased mitochondrial density in trigeminal nucleus after bradykinin stimulation $p<0.001$
Jarvis et al. 1990	Rabbit corneal nociceptors (in vitro)<br>n = 20	632.5	5mW; pulse width 0-1, 800sec	300s LI 4mm diameter spot	Mechanical Single-fiber discharge in excised cornea Aδ & C fibers	No change after 120mins ?
Wakabayashi et al. 1993	Rat mandibular branch of trigeminal nerve (tooth incisor)<br>n = 12<br>(in vivo)	830	350mW, cw (direct to tooth)	120s LI 10mm above cervical surface of lower incisor	Electrical stimulation	Inhibition of **C** fiber EP spike activity; no change in A**δ** (persisted for 15 mins after LI) $p<0.005$

Kasai et al. 1994	Rabbit<br>sural nerve<br>n = 7<br>(in vivo—exposed nerve)	632.8	1mW, 100Hz	LI (duration not stated)<br>40mm above nerve in oil bath;<br>4mm diameter spot	Mechanical—pinch	Inhibition of pinch stimulation<br>& spontaneous neural discharge<br>$p<0.01$
Tsuchiya et al. 1994	Rat<br>saphenous nerve<br>n = 12<br>n = 7 (Rx'd with capsaicin) at birth which destroys Aδ & C fibers	830	40mW, cw	180s LI<br>PD: 1W/cm^2	Pinch, heat, cold, turpentine, brush—neuronal discharge measured at dorsal horn following LI	Decreased discharge of nociceptors (by ~30%)<br>Exp 1: pinch; $p<0.01$<br>Exp 2: heat; $p<0.01$<br>Exp 3: cold; $p<0.01$<br>Exp 4: inj turpentine $p<0.01$<br>Exp 5: brush; no change<br>Exp 6: in Capsaicin Rx'd rats—no change
Jimbo et al. 1998	Mice<br>axon of cultured DRG neuron<br>(patch clamp)	830	16.2mW, cw	LI 60s<br>ED: 1J/cm^2<br>area irrad: 75µm	Chemical<br>Bradykinin	Inhibition of EPs<br>2 mins after LI
Orchardson et al. 2000	Ferrets stimulation of exposed dentine in	i) 632.8 (aiming beam)	i) ~1mW	i) LI 60s<br><br>iia) & iib) 120s	Mechanical	i) No effect on intradental nerve responses

	canine teeth<br><br>(in vivo)	iia) 1064<br>iib) 1064	iia) 0.6 – 1.5W<br>iib) >.2W			iia) inc or dec intradental n response<br>iib) depressed intradental n response
