# Supplementary material for: A Translational Review of Mechanisms of Effectiveness of Photobiomodulation on Somatosensory Neurons and the Peripheral Nervous System—From Molecular Mechanisms to Clinical Applications in Medicine and Dentistry
Source: Curr Issues Mol Biol. 2026 Jul 9;48(7):695. doi: 10.3390/cimb48070695 (PMC13409449; doi:10.3390/cimb48070695)
Supplement: Supplementary file 1 [file cimb-48-00695-s001.zip › Supplementary Material D dental sys rev 22-6-26 .pdf]

Supplementary Materials D: Systematic Reviews of Photobiomodulation in Oral and Dental Pain Conditions.

Condition	Author	Year	Principal Finding
Burning Mouth Syndrome	Hanna et al.; Khemiss et al.	2021–2024	PBM reduced symptoms and pain.
Temporomandibular Disorders	Petrucci et al.; Maia et al.; Melis et al.; Herpich et al.; Munguia et al.; Xu et al.; Candido-do-Prado et al.; Diaz et al.	2011–2025	Consistent evidence supporting pain reduction.
Dental Injection Pain	Kulkarni et al.; Altuhafy et al.; Hakimiha et al.; Amrollahi et al.; Shekarchi et al.	2022–2025	Reduced pain during dental anaesthetic injections.
Third Molar Surgery	Brignardello-Petersen et al.; Sourvanos et al.	2012 - 2023	Reduced postoperative pain, swelling and trismus.
Orthodontic Pain	Ren et al.; Sonesson et al.	2015-2016	Reduced orthodontic pain.
Oral Mucositis	Bjordal et al.; Parra-Rojas et al.; Bensadoun et al.	2011–2025	Preventive and therapeutic benefits demonstrated.
